# *Clostridioides difficile*’s virulence requires efficient holin-mediated toxin secretion

**DOI:** 10.1016/j.isci.2025.112586

**Published:** 2025-05-07

**Authors:** Nicholas V. DiBenedetto, Marine Oberkampf, Aline Crouzols, Laura Cersosimo, Vladimir Yeliseyev, Lynn Bry, Johann Peltier, Bruno Dupuy

**Affiliations:** 1 Massachusetts Host-Microbiome Center, Department Pathology, Brigham and Women’s Hospital, Harvard Medical School, Boston, MA, USA; 2 Department of Molecular Biology and Microbiology, Tufts University Medical Center, Boston, MA, USA; 3 Pathogenesis of Bacterial Anaerobes, Department of Microbiology, Institut Pasteur, Université Paris-Cité, UMR-CNRS 6047 Paris, France; 4 Institute for Integrative Biology of the Cell (I2BC), CEA, CNRS, Université Paris-Saclay, Gif-sur-Yvette, France

**Keywords:** Bacteriology, Biological sciences, Microbiology, Natural sciences

## Abstract

The human pathogen *Clostridioides difficile* causes pseudomembranous colitis through release of the potent TcdA and TcdB toxins. These toxins lack canonical N-terminal secretion signal sequences for translocation across the bacterial membrane, suggesting alternate mechanisms of release including targeted secretion mediated by the holin-like protein TcdE and passive release from cell lysis. Here, we show how the lack of the TcdE holin profoundly reduces toxin secretion in the high toxin-producing strains UK1 and VPI10463, independently of cell lysis. Remarkably, *tcdE* deletion in either strain rescued highly susceptible gnotobiotic mice from lethal infection. Δ*tcdE*-infected mice demonstrated undetectable levels of toxin acutely and long-term survival, despite active toxin gene expression and cytoplasmic accumulation in mutant strains. These findings demonstrate the dominant role of the TcdE holin in toxin secretion *in vivo* and host disease and confirm the use of a conserved and non-lytic holin-mediated toxin secretion mechanism among toxigenic species of *Clostridia*.

## Highlights

• TcdE holin-like protein enables *C. difficile* toxin secretion without cell lysis
• *tcdE* deletion prevents toxin secretion despite active *tcdA*/*tcdB* transcription
• Δ*tcdE* mutants accumulate toxin inside cells but fail to cause disease in mice
• Targeting TcdE as a therapeutic target may prevent severe *C. difficile* infections

## Graphical abstract

**Figure:**
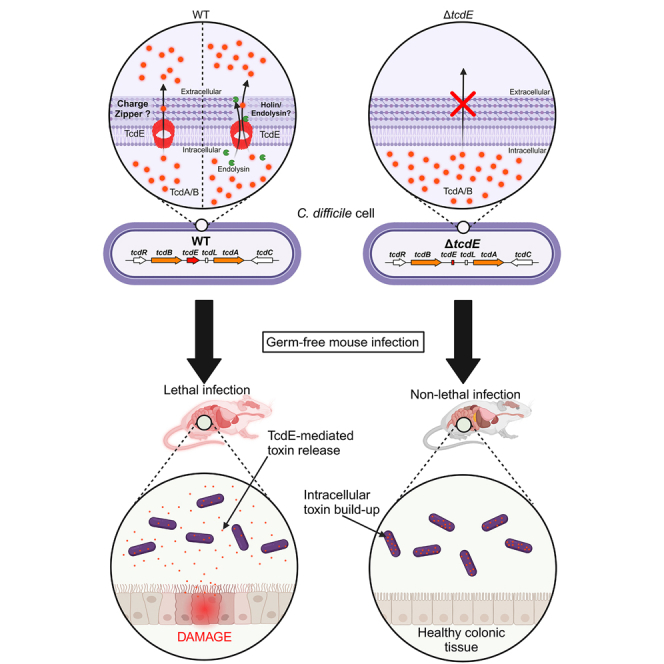

## Introduction

Exotoxin release from bacterial pathogens commonly occurs via specialized protein secretion systems. Toxigenic bacteria harbor diverse secretion systems to facilitate toxin release from the cytoplasm.^1^ The structures of these systems vary relative to the membrane and cell wall components that need to be traversed in Gram-positive versus Gram-negative bacteria. Gram-positive bacteria, which lack an outer membrane, use two main pathways for protein secretion, namely the general secretory pathway (Sec) and the twin-arginine translocation pathway (Tat).^2^ Proteins using these systems contain an N-terminal secretion signal recognition sequence for membrane passage and release.^3^ In contrast to other bacterial toxins, large clostridial toxins (LCTs) lack predictable secretion signals. LCTs form a family of bacterial exotoxins that exceed 200 kDa in size, and that inactivate host small GTPases to disrupt the actin cytoskeleton and promote host cell death.^4^^,^^5^ In addition to *C. difficile* toxins TcdA and TcdB, the LCT family includes *Clostridium novyi* toxin TcnA, *Clostridium perfringens* toxin TpeL, and *Paeniclostridium sordellii* toxins TcsL and TcsH. Recent studies in *C. difficile*, *P. sordellii*, and *C. perfringens* suggest that LCT secretion may leverage a conserved, non-lytic, holin-mediated transport mechanism.^6^^,^^7^^,^^8^^,^^9^

**Figure 1:**
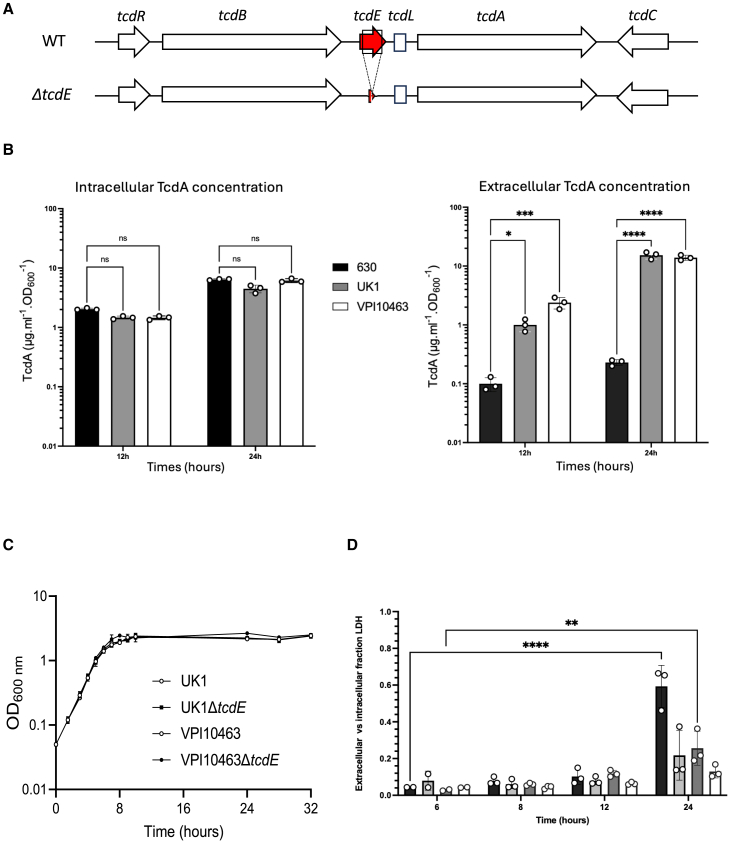
Toxin release occurs independently of cell lysis in *C. difficile* strains VPI10463 and UK1 (A) Schematic representation of the *tcdE* genetic environment and *tcdE* deletion (Δ*tcdE*). (B) TcdA titers in extracellular and intracellular fractions of 630Δ*erm*, VPI10463 and UK1 strains after 12 and 24 h of growth. The strains were grown in TY medium and TcdA was quantified using TcdA-ELISA. Means and SD are shown; *n* = 3 independent experiments. ∗*p* ≤ 0.05, ∗∗∗*p* ≤ 0.001 and ∗∗∗∗*p* ≤ 0.0001 by a one-way ANOVA. (C) Growth curves of VPI10463 and UK1 strains, and their respective Δ*tcdE* mutants in TY medium. Means and SD are shown; *n* = 3 independent experiments. (D) Ratio of the lactate dehydrogenase (LDH) activity in the supernatants (extracellular fraction) and the cell lysates (intracellular fraction) of VPI10463 and UK1 strains and their respective Δ*tcdE* mutants used as an indicator of autolysis. LDH activity was measured using the CytoTox 96 Non-Radioactive Cytotoxicity Assay (Promega). Means and SD are shown; *n* = 3 independent experiments. ∗∗*p* ≤ 0.01 and ∗∗∗∗*p* ≤ 0.0001 by a two-way ANOVA followed by a Dunnett’s multiple comparison test. Extracellular and intracellular LDH activity values used to determine the ratio of LDH are presented in Figure S3. Means and SD are shown; *n* = 3 independent experiments. ∗∗∗∗*p* ≤ 0.0001 by a one-way ANOVA.

*C. difficile’s* Pathogenicity Locus (PaLoc) encodes the *tcdA* and *tcdB* genes and three accessory genes: *tcdR, tcdE*, and *tcdC* (Figure 1A).^10^ TcdR is an alternative sigma factor required for PaLoc gene expression, while TcdC has been proposed to negatively regulate transcription as an anti-sigma factor.^11^^,^^12^^,^^13^^,^^14^^,^^15^ TcdE shares homology with bacteriophage holins and has been shown to promote toxin release while its role was somehow controversial.^8^^,^^16^ Holins are small, bacteriophage-encoded membrane proteins that promote bacteriophage release. They oligomerize to form pores in the cytoplasmic membrane, supporting the export of bacteriophage-encoded endolysins which digest the peptidoglycan cell envelope and, in the process, can promote cell lysis.^17^

In addition to the non-lytic TcdE-dependent translocation of the PaLoc toxins,^8^ lytic release of toxins in stationary phase can occur in the laboratory strain 630Δ*erm* of *C. difficile* via activity of the lytic peptidoglycan transglycosylase Cwp19,^18^ suggesting that lytic and non-lytic mechanisms may coexist and function during different phases of cell growth and relative to stressors encountered *in vivo.*^19^ Both systems could act in other toxigenic *Clostridia* that use holin-like proteins for LCT release as well, such as TpeL in *C. perfringens* and TcsL in *P. sordellii*,^6^^,^^7^^,^^8^^,^^9^ as they both harbor Cwp19 homologs, further supporting functions of these systems in *Clostridial* toxin release.

Within this framework, we hypothesized that the TcdE holin mediates efficient toxin release during periods of high toxin expression, with lytic mechanisms providing a default pathway integrated with physiologic and stress responses inducing pathogen cell lysis.^16^ We investigated which secretion mechanisms promoted PaLoc encoded toxin release in *C. difficile* by creating in-frame Δ*tcdE* mutants in high toxin-producing strains, evaluating their functions *in vitro* in toxin release, and disease course and severity in a gnotobiotic mouse model. Our findings show how the TcdE holin strongly impacts toxin release in a manner that functions independently of bacteriolysis. *In vivo,* we illustrate how the TcdE holin promotes symptomatic disease in highly vulnerable gnotobiotic mice, by enabling toxin release to damage mucosal surfaces. Our findings confirm the function of this secretion system in Gram-positive toxigenic species that leverages holin-like proteins to secrete LCGTs.

## Results

### High toxin-producing strains of *C. difficile* possess an efficient secretion system

We evaluated TcdE’s functions on toxin release *in vitro* and *in vivo* in the high-toxin producing and clinical strains VPI10463 and ribotype 027 epidemic strain UK1.^20^^,^^21^ Both strains cause symptomatic infections in conventional and defined-association mouse models, resulting in severe disease and death.^22^^,^^23^ We first compared intracellular and extracellular concentrations of TcdA in these strains and in the low toxin-producing strain 630Δ*erm* at 12 and 24h of growth in tryptone yeast extract (TY) broth, a medium that supports high toxin expression.^24^^,^^25^ The VPI10463 and UK1 strains produced higher toxins level than the 630Δ*erm* strain (Figure 1B). Intracellular concentrations of TcdA in strain 630Δ*erm* are similar to those of VPI10463 and UK1 at 12 and 24 h. In contrast, extracellular concentrations of TcdA were 10- and 20-fold higher in UK1 and VPI10463, respectively, at 12h, and 10-fold for both strains at 24h, compared to strain 630 (Figure 1B). These findings illustrate that the high toxin-producing and virulent strains VPI10463 and UK1 released the majority of toxins that they produce, while toxin mainly remained in the intracellular compartment of the less virulent and low toxin-producing 630Δ*erm* strain (Figure S1A). Comparable phenotypes were identified in additional clinical strains E25 and CD21-013, known as low and high toxin producers, respectively (Table S1; Figures S1B and S1C).

### Toxin A and B secretion in VPI10463 and UK1 depends upon TcdE but not TcdL

We generated in-frame *tcdE* deletion mutants to further evaluate TcdE’s functions in toxin secretion in VPI10463 and UK1 (Figures 1A and S2A). The wild-type and isogenic mutant strains grew similarly in TY medium (Figure 1C). The low extracellular/intracellular ratio of lactate dehydrogenase (LDH) activity, a cytoplasmic enzyme used to monitor cell lysis, confirmed nominal cellular lysis between the wild-type and *tcdE* mutant strains in the first 12h of growth (Figures 1D and S3). However, the LDH ratio increased significantly at 24h in the UK1 and VPI10463 parental strain, but to a much lesser extent in the *tcdE* mutants suggesting that TcdE activity can contribute in part to cell lysis during late stationary phase.

**Figure 2:**
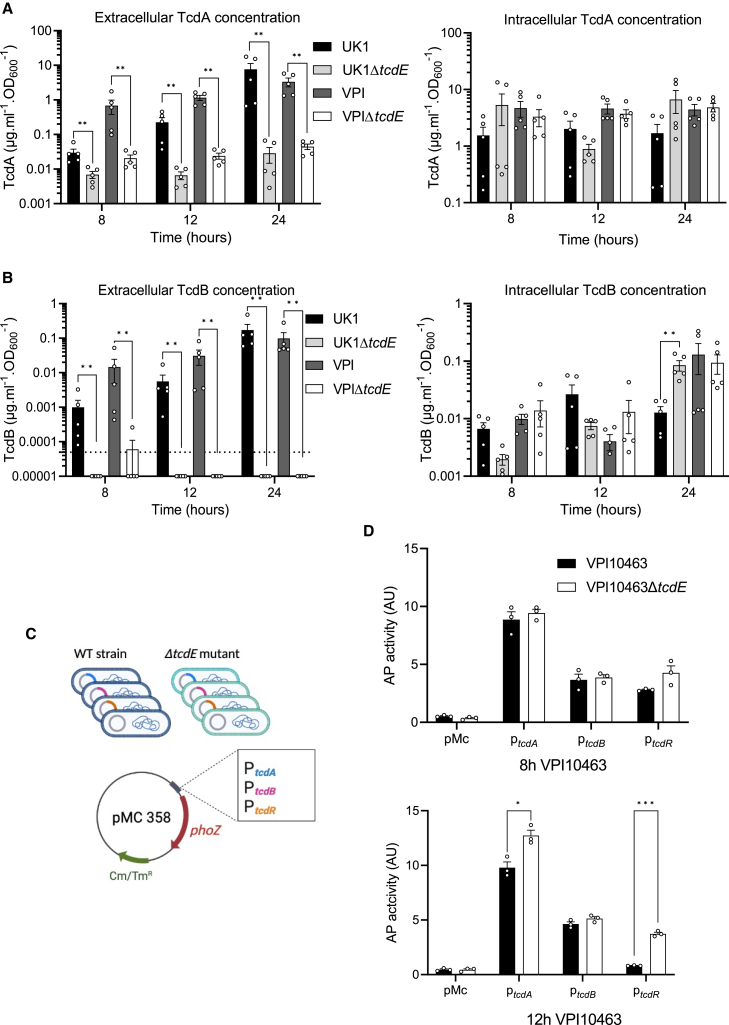
TcdE mediates TcdA and TcdB release in VPI10463 and UK1 strains *in vitro* TcdA (A) and TcdB (B) titers in extracellular (left panel) and intracellular (right panel) fractions of VPI10463 and UK1 strains and their respective Δ*tcdE* mutants, after 8, 12 and 24 h of growth. Strains were grown in TY medium, and toxins were quantified using TcdA- and TcdB-ELISA. Means and SEM are shown; *n* = 5 independent experiments ∗∗*p* < 0,01 by a Mann-Whitney test. Horizontal dotted line shows thresholds of detection. (C) Schematic representation of transcriptional fusions constructions. Transcriptional fusions of promoter regions of approximately 500 bp of *tcdA*, *tcdB* or *tcdR* genes fused to the reporter gene *phoZ*, were introduced by conjugation into the VPI10463 wild-type strain and the isogenic Δ*tcdE* mutants. (D) Alkaline phosphatase (AP) activity of P*tcdA*:*phoZ*, P*tcdB*:*phoZ*, and P*tcdR*:*phoZ* fusions expressed from pMC358 in VPI10463 and VPI10463Δ*tcdE*. Strains were grown in TY medium and samples assayed for AP activity were collected at 8 and 12h of growth. Means and SEM are shown; *n* = 3 independent experiments. ∗*p* ≤ 0.05 and ∗∗∗*p* ≤ 0.001 by an unpaired t test.

Extracellular and intracellular toxin levels measured at 8, 12, and 24h of growth showed dramatic reductions in extracellular TcdA and TcdB in the Δ*tcdE* mutants of UK1 and VPI10463 (Figures 2A and 2B). Strikingly, extracellular TcdB levels fell below the limit of detection in the Δ*tcdE* mutants. Conversely, intracellular TcdA and TcdB accumulation remained comparable between the Δ*tcdE* and the wild-type strains (Figures 2A and 2B). To evaluate if *tcdE* deletion affected toxin gene transcription, plasmids carrying transcriptional fusions of the *tcdA*, *tcdB*, and *tcdR* promoter regions with the alkaline phosphatase (AP) gene *phoZ*^26^ were transferred into the *tcdE* mutant and wild-type strains (Figure 2C). AP activities at 8h and 12h of growth were comparable in the VPI10463 and the isogenic *ΔtcdE* strains*,* with a slight increase in *tcdR* and *tcdA* transcription at 12h (Figure 2D)*.* The same trend was observed in the wild-type and isogenic Δ*tcdE* strains of UK1 carrying the transcriptional fusions (Figure S4). qRT-PCR analyses of *tcdA*, *tcdB*, and *tcdR* transcription in VPI10463 strain versus its isogenic *tcdE* mutant further showed minor or inconsequential changes in PaLoc gene expression (Figure S5), confirming that *tcdE* mutation had no deleterious effects on toxin gene expression. These data highlight TcdE’s crucial role in toxin secretion in such high toxin producing strains and demonstrate that it functions in a cell lysis–independent manner.

Similar to the *Clostridial* LCT loci, T10SS within Gram-negative bacterial species loci encode secreted target proteins within the same genetic locus as the holin/endolysin pair,^27^ suggesting potential for the TcdE holin to act in a comparable manner with an as-yet unidentified endolysin. Within the *C. difficile* PaLoc, an endolysin gene remnant, *tcdL*, occurs in most strains.^28^ However, an intact version of the TcdL endolysin has been identified in cryptic clades of *C. difficile* that carry a Mono-Toxin B Paloc.^29^^,^^30^ These findings raise the interesting question as to whether *C. difficile* uses the TcdL remnant endolysin with TcdE to facilitate PaLoc toxin release. We evaluated the role of TcdL in toxin secretion by generating an in-frame *tcdL* deletion mutant in VPI10463 (Figures S2B and S6A). The Δ*tcdL* mutant grew similarly to the wild-type and the *tcdE* deletion mutant strains in TY medium (Figure S6B). While extracellular levels of TcdA and TcdB were dramatically reduced in the Δ*tcdE* mutant, levels of both toxins remained similar between the Δ*tcdL* mutant and wild-type strains (Figures S6C and S6D), indicating that TcdE does not require the putative TcdL endolysin fragment to facilitate *C. difficile* toxin secretion.

The ribotype 027 strain UK1 produces an additional toxin, the binary cytolethal distending toxin (CDT), encoded in the Cdt toxin locus or CdtLoc, by genes unlinked to the PaLoc.^31^ In agreement with a previous report,^32^ SignalP^33^ analyses identified putative Sec signal peptides in the N-terminus of the CDT subunits CdtA and CdtB, suggesting their release by a holin-independent manner (Figures S7A and S7B). Consistent with this prediction, we found that *tcdE* deletion had no impact on CdtB secretion in the UK1 strain (Figure S7C). Moreover, CdtB secretion occurs jointly after production, as shown by the limited pools of intracellular toxin (Figure S7C), in contrast to TcdA and TcdB, which must first accumulate in the cytosol prior to TcdE-mediated secretion once they have reached a critical threshold (Figures 2A and 2B). These findings confirm specificity of the TcdE holin for the PaLoc TcdA and TcdB toxins and use of a putative Sec signal peptide in the CdtB toxin release.

### TcdE inactivation prevents toxin secretion and host disease *in vivo*

**Figure 3:**
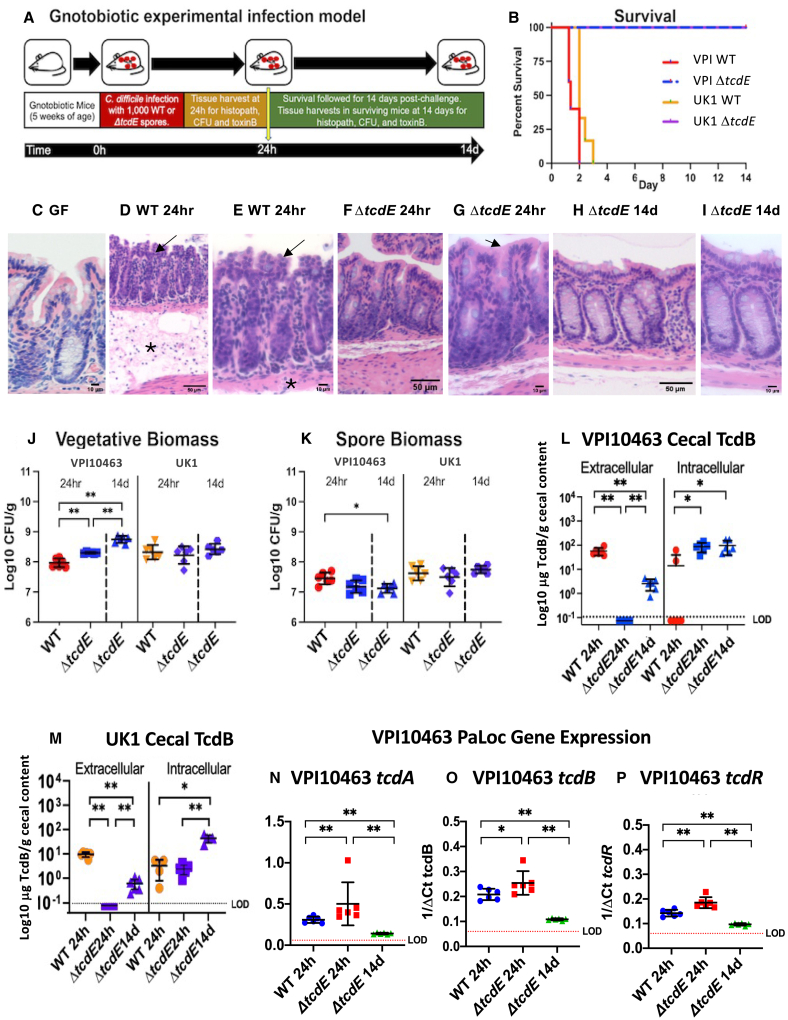
TcdE deletion rescues gnotobiotic mice from lethal *C. difficile* infection (A) Experimental overview of the gnotobiotic infection model. (B) Survival of mice challenged with wild-type VPI10463 (red), VPI10463*ΔtcdE* (blue), UK1 (orange) or UK1Δ*tcdE* (purple), *n* = 10 mice per strain. (C–H) Hematoxylin and eosin (H&E) sections of the colonic mucosa. (C) Germ-free GF mouse at 400× magnification showing intact epithelium and normal lamina propria cellularity. (D) Representative GF mouse infected with wild-type VPI10463 at 24h post-challenge, 100× magnification showing severe mucosal damage with transmural inflammation, tissue edema (asterix) and formation of pseudomembranes above the surface epithelium. (E) Inset from panel D at 200× magnification showing transmural neutrophilic infiltrates as well as immune cells in sub-mucosal blood vessels, at the bottom. (F) Colon at 200× magnification of Δ*tcdE*-infected mouse showing limited surface epithelial ruffling but intact epithelium and with nominal inflammatory infiltrates at 24 h post-challenge, in contrast to mice infected with the wild-type strain (panels D-E). (G) 400× magnification of surface colonic epithelium from Δ*tcdE* infected mouse showing focal ruffling of surface colonocytes but without formation of pseudomembranes. (H) 400× magnification of colonic mucosa at 14 days in a surviving Δ*tcdE*-infected mouse showing intact colonic epithelium and limited lymphocytic infiltrates in the lamina propria. (I) *C. difficile* vegetative biomass in cecal contents from mice infected with VPI10463 and UK1 or the isogenic Δ*tcdE*-mutant strains at 24h and from the Δ*tcdE*-infected mice at 14 days, *n* = 6 mice per strain. Significance values for non-parametric Kruskal-Wallis test shown are ∗∗*p* = 0.0022. (J) *C. difficile* spore biomass in cecal contents from mice infected with VPI10463 and UK1 or the isogenic Δ*tcdE*-mutant strains at 24h and from the Δ*tcdE*-infected mice at 14 days, *n* = 6 mice per strain. Significance values for non-parametric Kruskal-Wallis test shown are ∗*p* = 0.012. (K) Cecal extracellular and intracellular toxin B levels from mice infected with wild-type VPI10463 or the isogenic Δ*tcdE*-mutant strains at 24h and from the Δ*tcdE*-infected mice at 14 days. Dotted line with LOD indicates limit of detection, *n* = 6 mice per strain. Significance values for non-parametric Kruskal-Wallis test shown are ∗∗*p* = 0.0022 and ∗*p* = 0.012. (L) Data comparable to panel k for strain UK1, *n* = 6 mice per strain. (M–O) qRT-PCR of cecal *tcdA*, *tcdB*, and *tcdR* expression in mice infected with the wild-type or Δ*tcdE* VPI10463 strains at 24 h and surviving Δ*tcdE* strain-infected mice at 14 days, *n* = 6 mice per strain. Bars show mean and standard deviation. Kruskal-Wallis significance values as in panels k and l.

To evaluate the impact of *tcdE* deletion on *C. difficile’s* virulence *in vivo*, 5-week-old germfree mice were infected with the Δ*tcdE* mutants or corresponding, isogenic wild-type strains (Figure 3A). Mice infected with 1000 spores of wild-type VPI10463 rapidly succumbed to infection over 48–72h (Figure 3B) with symptoms of lethargy, weight loss (Figure S8A), and diarrhea developing at 20h post-challenge. By 24h, infected mice demonstrated transmural inflammation with pseudomembrane formation and substantive mucosal edema when compared to the colons of uninfected germ-free mice (compare Figure 3C, with Figures 3D and 3E). In contrast, all mice infected with the isogenic Δ*tcdE* strain survived infection (Figure 3B). Δ*tcdE*-infected mice demonstrated mild to subclinical symptoms of infection with no loss of weight (Figure S8A). The colonic mucosa of Δ*tcdE-*infected mice demonstrated focal areas of mild apical epithelial ruffling at 24h post-challenge but without broad epithelial destruction and associated transmural inflammation (Figures 3F–3I). By 14 days post-challenge, Δ*tcdE-*infected mice demonstrated intact colonic epithelium with mild lymphocytic infiltrates in the mucosa (Figures 3H and 3I). Mice infected with the wild-type and Δ*tcdE* mutant strains of UK1 similarly showed 100% rescue from lethal infection with the Δ*tcdE* mutant strain (Figure 3B). Clinically, mice infected with the Δ*tcdE* mutant of UK1 showed no weight loss (Figure S8B) and demonstrated comparable histopathologic findings in the colonic mucosa to mice infected with Δ*tcdE* mutant of VPI10463 up to 14 days (Figures S8C–S8E).

The vegetative biomass of VPI10463 Δ*tcdE* was 2-fold higher than that of the wild-type VPI10463 at 24h post-challenge and increased 5-fold from 24h to 14 days in Δ*tcdE-*infected mice (Figure 3J). In contrast, spore biomass remained equivalent between the strains, including at 14 days in the *tcdE* mutant (Figure 3K). In UK1-infected mice, vegetative and spore biomass remained comparable between the wild-type and the Δ*tcdE* strain over time (Figures 3J and 3K). These data indicate that the Δ*tcdE* strains have comparable or slightly better fitness to colonize the gnotobiotic mouse intestine than the corresponding wild-type strains.

At 24h of infection, while VPI10463-infected mice demonstrated high levels of TcdB toxin, levels fell below detectable thresholds in mice infected with the isogenic *tcdE* mutant (Figure 3L). By 14 days post-infection, low toxin B levels were detectable in surviving Δ*tcdE-*infected mice, suggesting release of toxin by a TcdE-independent mechanisms such as via cellular lysis, but remained 20-fold lower than the acute levels seen at 24h in mice infected with the wild-type strain (Figure 3L). In contrast, intracellular TcdB levels were slightly increased in VPI10463 Δ*tcdE* at 24h post-infection when compared to the isogenic wild-type and remained constant over time in the mutant strain (Figure 3L). The same variations of intra- and extracellular levels of TcdB were observed in mice infected with the UK1 and UK1 Δ*tcdE* strains (Figure 3M). *tcdE* deletion did not impact *in vivo* on toxin gene expression as qRT-PCR of *tcdA*, *tcdB*, and *tcdR* transcripts in VPI10463 (Figures 3N–3P, respectively) showed slightly elevated expression of *tcd* genes in the Δ*tcdE* mutant at 24h of infection compared to the wild-type strain. Although intracellular level of toxin remained high in Δ*tcdE* mutant-infected mice (Figure 3L), expression levels of toxin genes in the Δ*tcdE* mutants fell 2 to 5-fold by 14 days (Figures 3N–3P). We observed comparable effects of *tcdE* deletion on toxin gene expression *in vivo* at 24h with UK1 and its isogenic *tcdE* mutant (Figures S8F–S8H). However, unlike VPI10463, toxin gene expression levels increased at 14 days in UK1 Δ*tcdE* mutant-infected mice (Figures S7F–S7H), with the colonic epithelium remaining intact (Figure S8E), reinforcing the impact of *tcdE* deletion on toxin secretion during *in vivo* infection.

## Discussion

Our findings demonstrate the profound effects of *C. difficile’s* TcdE holin on host outcomes from infection through its central role in facilitating extracellular toxin release. The undetectable levels of extracellular toxin acutely in Δ*tcdE* mutant-infected mice and limited mucosal damage supported that toxin release from alternate mechanisms, including cellular lysis via Cwp19 or other lytic mechanisms, was irrelevant. By 14 days post-challenge, while extracellular toxin levels rose slightly, they remained 20-fold lower than those seen at 24h in infection with the wild-type strains, suggesting that alternate mechanisms of toxin release play a limited role *in vivo* for high toxin-producing strains, particularly during acute host infection.^18^

While the TcdE holin mediates toxin release, how it facilitates release remains an interesting question. In the type 10 secretion systems (T10SS) characterized in Gram negative species, holins have been shown to promote protein secretion through novel mechanisms that use a holin/endolysin pair.^34^ The T10SS holins oligomerize to form pores in the cytoplasmic membrane, enabling the transport and release of a cytosolic endolysin into the periplasm. The peptidoglycan hydrolase activity of the endolysin locally permeabilizes the cell wall to allow specific protein substrates to be transported through the peptidoglycan layer and secreted.^34^ The *Serratia marcescens* chitinases leverage this mechanism, via translocation of the endopeptidase endolysin ChiX into the periplasmic space via the holin ChiW.^35^ Similarly, the secretion of the unusual typhoid AB-toxin of *Salmonella enterica typhi* requires peptidoglycan remodeling by muramidase activity of endolysin TtsA, which is believed to be translocated across the internal membrane by an unidentified holin.^36^

Our analyses in isogenic mutant strains indicated that TcdE does not require the putative TcdL endolysin fragment to facilitate *C. difficile* toxin secretion, leading us to believe that the endolysin partner may be located elsewhere in the genome. However, we cannot exclude the possibility that the remnant endolysin TcdL may lack hydrolysis activity but could still mediate aspects of toxin transport if were to instead interact directly with the TcdB toxin rather than facilitate release through interactions with TcdE.^37^

Though LCTs use a holin/endolysin pair to traverse the peptidoglycan layer in *Clostridia*, how they cross the cell membrane remains ill-defined, given their lack of an N-terminal signal sequence. A recent study by Saadat and Melville suggest that *C. perfringens’* LCT toxin TpeL can be directly transported by its TpeE holin using a charge zipper mechanism. After insertion into the membrane, TpeL induces folding and oligomerization of TpeE around it, to form a pore that facilitates subsequent secretion.^7^ In the case of *C. difficile*, elucidation of the essential function of the TcdE holin in PaLoc toxin release opens opportunities to further resolve mechanisms of toxins transport through the cellular membrane and peptidoglycan layers, including requirements for local peptidoglycan remodeling by chromosomal genes. Our findings identify TcdE as a central target of vulnerability in *C. difficile’s* PaLoc toxin release system, particularly during acute infections with high toxin-producing and epidemic strains, and support therapeutic interventions that reduce its expression and function *in vivo.*

### Limitations of the study

While these studies establish a critical role for the TcdE holin-like protein in facilitating non-lytic secretion of *C. difficile* toxins, several limitations remain. The precise molecular mechanism by which TcdE mediates toxin translocation across the cytoplasmic membrane is still unclear, particularly given the absence of a defined endolysin partner. Although the putative endolysin gene *tcdL* was found to be dispensable for toxin release, the identity and function of any alternative peptidoglycan-modifying factors remain unresolved. *In vivo* studies were performed exclusively in gnotobiotic mice, which lack a complex microbiota; it remains unknown whether, in a more clinically relevant conventional mouse model, microbiota-driven inflammation or host immune responses might promote cell lysis and restore some level of toxin release in the absence of TcdE, potentially altering disease outcomes compared to the gnotobiotic mouse model. Finally, while the results were consistent across experiments, group sizes were limited to six mice per strain across two independent studies. Greater confidence in the findings could be achieved with larger cohorts (e.g., ≥10 mice) and replication in at least three independent experiments.

## Resource availability

### Lead contact

Further information and requests for resources and reagents should be directed to and will be fulfilled by the lead contact, Bruno Dupuy (bdupuy@pasteur.fr).

### Materials availability

Strains generated in this study are available from the lead contact without restrictions.

### Data and code availability

• All data reported in this paper will be shared by the lead contact upon request.
• This paper does not report original code. Any additional information required to reanalyze the data reported in this paper is available from the lead contact upon request.

## Acknowledgments

This work was funded by the Institut Pasteur and the “Integrative Biology of Emerging Infectious Diseases” (LabEX IBEID) funded in the framework of the French Government’s “Programme Investissements d’Avenir” to B.D; the ANR-20-CE15-0003 (Difficross) to J.P; and grants R01AI153605, R01AI179807 and P30DK34854 from the National Institute of Health in the United States, and a capital grant from the 10.13039/100011495Massachusetts Life Sciences Center to LB.

## Author contributions

B.D., J.P., M.O., N.V.D., and L.B. contributed to the experiment design and interpreted all the results. M.O., J.P., A.C., and B.D. created bacterial strains and performed *in vitro* assays (AP, LDH, and toxins) and qRT-PCR. N.V.D., L.C., V.Y., and L.B. carried out mice survival studies, gut analyses for bacterial biomass, toxin levels, qRT-PCR gene expression, and histopathologic analyses. B.D., J.P., and L.B. contributed to writing the first draft of the manuscript and all of the authors commented on manuscript drafts.

## Declaration of interests

The authors declare no competing interests.

## STAR★Methods

### Key resources table

**Table:** 

REAGENT or RESOURCE	SOURCE	IDENTIFIER
**Bacterial strains**		

*E. coli* NEB10β	New England Biolabs	C3019H
*E. coli* HB101(RP4)	Laboratory stock	N/A
*E. coli* ER3413	Laboratory stock	CP009789.1
*E. coli* HB101(RP4) + pDIA7080	This paper	EC1307
*E. coli* HB101(RP4) + pDIA7090	This paper	EC1383
*E. coli* ER3413 + pDIA7296	This paper	EC1659
*C. difficile* 630Δ*erm*	Laboratory stock, Hussain et al.^38^	N/A
*C. difficile* VPI10463	Laboratory stock, Hammond and Johnson^20^	N/A
*C. difficile* UK1	Laboratory stock, Killgore et al.^21^	N/A
*C. difficile* E25	Laboratory stock, Kurka et al.^39^	N/A
*C. difficile* CD21-013	Laboratory stock	N/A
*C. difficile* VPI10463 Δ*tcdE*	This paper	CDIP1688
*C. difficile* UK1 Δ*tcdE*	This paper	CDIP1753
*C. difficile* VPI10463 carrying pMC358	This paper	CDIP1855
*C. difficile* VPI10463 ΔtcdE carrying pMC358	This paper	CDIP1920
*C. difficile* UK1 carrying pMC358	This paper	CDIP1838
*C. difficile* UK1 Δ*tcdE* carrying pMC358	This paper	CDIP1854
*C. difficile* VPI10463 ΔtcdL	This paper	CDIP2239
*C. difficile* VPI10463 carrying p112	This paper	CNRS_CD166
*C. difficile* VPI10463 ΔtcdE carrying p112	This paper	CNRS_CD170
*C. difficile* VPI10463 carrying p113	This paper	CNRS_CD167
*C. difficile* VPI10463 ΔtcdE carrying p113	This paper	CNRS_CD171
*C. difficile* VPI10463 carrying p115	This paper	CNRS_CD169
*C. difficile* VPI10463 ΔtcdE carrying p115	This paper	CNRS_CD172
*C. difficile* UK1 carrying p112	This paper	CDIP1834
*C. difficile* UK1 Δ*tcdE* carrying p112	This paper	CDIP1850
*C. difficile* UK1 carrying p113	This paper	CDIP1837
*C. difficile* UK1 Δ*tcdE* carrying p113	This paper	CDIP1853
*C. difficile* UK1 carrying p115	This paper	CDIP1836
*C. difficile* UK1 Δ*tcdE* carrying p115	This paper	CDIP1852

**Oligonucleotides**		

See Table S1		

**Recombinant DNA**		

pMSR0	Peltier et al.^40^	N/A
pMC358	Edwards et al.^26^	N/A
pDIA7080: pMSR0 derivative for *tcdE* deletion in VPI10463	This paper	N/A
pDIA7090: pMSR0 derivative for *tcdE* deletion in UK1	This paper	N/A
pDIA7296: pMSR0 derivative for *tcdL* deletion in VPI10463	This paper	N/A
p112: pMC358 derivative for P_*tcdR*_-*phoZ* fusion	This paper	N/A
p113: pMC358 derivative for P_*tcdB*_-*phoZ* fusion	This paper	N/A
p115: pMC358 derivative for P_*tcdA*_-*phoZ* fusion	This paper	N/A

### Experimental model and study participant details

#### Strains, media and growth conditions

*C. difficile* strains described in the key resources table were grown in a Freiter’s chamber (Jacomex) under anaerobic atmosphere (5% H_2_, 5% CO_2_, and 90% N_2_) in TY^24^ or Brain Heart Infusion (BHI, Difco) media. When appropriate, cefoxitin (Cfx, 25μg/ml) and D-cycloserine (Cs, 250 μg/ml) and thiamphenicol (Tm, 7.5 μg/ml) were added to the culture medium. *E. coli* strains were grown in LB broth, and when necessary, ampicillin (Ap, 100 μg/ml) or chloramphenicol (Cm, 15 μg/ml) was added to the culture medium. Growth curves were obtained by monitoring OD_600_ at each time point from an overnight culture diluted to a starting OD_600_ of 0.05. For *in vivo* quantitation of total biomass of *C. difficile*, mouse cecal contents were collected into pre-weighed Eppendorf tubes with 0.5mL of pre-reduced PBS with 40mM cysteine (Millipore-Sigma) as a reducing agent. Tubes were weighed after adding material and transferred into a Coy anaerobic chamber (Coy Labs) at 37°C for serial dilutions with plating to selective *C. difficile* CHROMID agar (Biomérieux) or Brucella agar (Becton Dickinson) for commensal quantitation. *C. difficile* colonies were counted at 48 h of incubation. *C. difficile* spore preparations and counts were defined by exposing pre-weighed material to 50% ethanol for 60 minutes followed, by serial dilution and plating to *C. difficile* CHROMID agar. Vegetative cell biomass was calculated by subtracting the spore biomass from the total biomass and normalizing to the cecal mass.

#### Germ-free mouse infection studies

Animal studies were conducted in negative pressure BL-2 gnotobiotic isolators (Class Biologically Clean, Madison, WI)^41^ in the Massachusetts Host-Microbiome Center, under an approved institutional IACUC protocol. Equal ratios of 5 week-old male and female gnotobiotic mice were singly housed and challenged with 1x10^3^ of wild-type or Δ*tcdE C. difficile* spores. Progression of disease was assessed via body condition scoring.^42^ Mice were sacrificed at a BCS of 2-, or at defined timepoints at either 24 h or 14 days post-*C. difficile* challenge. Death was not used as an endpoint for studies. Cecal contents were collected for CFU enumeration, toxin ELISA, and qRT-PCR. The gastrointestinal tract and internal organs were fixed in zinc-buffered formalin (Z-FIX, Thermo-Fisher, Waltham, MA) for histopathological assessments.

### Method details

#### Plasmid and strain construction

All primers used are listed in Table S1. The Δ*tcdE* mutants in the *C. difficile* VPI10463 and UK1 backgrounds were obtained by allele-coupled exchange using the pMSR0 pseudo-suicide plasmid.^40^ Briefly, homology arms of both up- and downstream locations of the target gene were amplified by PCR from genomic DNA of *C. difficile* VPI10463 and UK1 strains and purified PCR products were directly cloned into the pMSR0 vector using NEBuilder HiFi DNA Assembly (New England Biolabs). The pMSR0-derived plasmids containing the allele exchange cassettes of the target genes were transformed into *E. coli* strain NEB10β (New England Biolabs) and verified by sequencing. Plasmids were then transformed into *E. coli* HB101 (RP4) and transferred by conjugation into appropriate *C. difficile* strains. *C. difficile* transconjugants were selected on BHI agar supplemented with cefoxitin, D-cycloserine and thiamphenicol. Single cross-over integrants were selected based on the size of the colonies after restreak of the transconjugants on BHI with thiamphenicol. Colonies that underwent the second recombination event were then selected on BHI plates containing anhydrotetracycline (ATc: 100 ng/ml). Growing colonies were then tested by PCR for the presence of the expected deletion. For construction of promoter::*phoZ* constructs, promoter regions of *tcdA*, *tcdB* and *tcdR* of approximately 500 bp were amplified by PCR and cloned into the linearized pMC358 vector.^26^

#### Alkaline phosphatase activity assays

*C. difficile* strains containing the *phoZ* reporter fusions were grown in TY medium at 37°C in anaerobic conditions from an overnight culture diluted to a starting OD_600_ of 0.05 and harvested at 8 and 12h growth after inoculation. Cell pellets were washed with 0.5 ml of cold Wash buffer (10 mM Tris-HCl, pH 8.0, 10 mM MgSO_4_), centrifuged and resuspended in 750 μl Assay buffer (1 M Tris-HCl, pH 8.0, 0.1 mM ZnCl_2_). 500 μl of the cell suspensions were transferred into separate tubes and mixed with 350 μl of Assay buffer and 50 μl of 0.1% SDS, before vortexing for 1 min. Sample tubes were incubated at 37°C for 5 min and then cooled on ice for at least 5 min. The assay starts by addition of 100 μl of 0.4% *p*NP (*p*-nitrophenyl phosphate in 1 M Tris-HCl, pH 8.0; Sigma-Aldrich) to each sample and incubation at 37°C. A sample without cell was prepared as a negative control. Upon development of a light-yellow color, the alkaline phosphatase reaction was stopped by addition of 100 μl of stop solution (1 M KH_2_PO_4_) and placing the tubes on ice. Time elapsed (min) for the assay was recorded. Samples were then centrifuged at max speed for 5 min and absorbance for each sample was read at both OD_420_ and OD_550_. Units of activity were calculated and normalized to cell volume by using the following formula: (OD_420_ − (1.75 × OD_550_) × 1000)/(*t* (min) ×  OD_600_ × vol. cells (ml)).

#### RNA extraction

Total RNAs were extracted from *C. difficile* strains grown in TY medium at 37°C in anaerobic conditions up to 8, 12 and 24 h. 10 ml of cultures were harvested and centrifuged (5000 rpm at 4°C) for 10 minutes. Supernatants were discarded and pellets immediately frozen and stored at -80°C. Pellets were resuspended in 1mL of RNApro (MP biomedicals) and transferred into Matrix B tubes for lysis by FastPrep (MP biomedicals) with 3x 40‘’ shaking cycle at speed 6.5, separated by 2 min incubation in ice. Tubes were then centrifuged 10 min at 13000 rpm at 4°C (conditions used for the entire procedure). Half a volume of chloroform was thereafter added to the cell lysates and were vortexed for 5 sec. After 10 min centrifugation the upper phases were collected, and nucleic acids were precipitated by addition of 500μl of cold ethanol and stored at -20°C for at least 30 min. After a 15 min centrifugation, pellets were washed 3 times with 500μl of cold 75% ethanol following by 5 min centrifugation. Ethanol was carefully removed and pellets air dried at room temperature and resuspended in 60 μl of RNase free water. RNAs were then treated by DNase (Turbo DNA free Kit) according to the manufacturer’s recommendations. *In vivo*, RNA was extracted from stools at 24h and 14 days on the King Fisher Flex instrument with the MagMax Microbiome Ultra Nucleic Isolation Kit (Thermo Scientific, Waltham, MA) and treated with DNase with the RNA Clean and Concentrator-5 kit (Zymo Research, Irvine, CA).

#### Reverse transcription and Real-time quantitative PCR

(i) From *in vitro* samples, RNA (1μg) was incubated 10 min at 70°C with 1μg of hexamer oligonucleotide primers (p(DN)6, Roche) before adding 10 μl of 5X AMV RT buffer, 4 μl of dNTP (25mM), 1μl of RNasine (Promega) and 1μl of AMV RT enzyme (Promega) for a 50μl reaction volume. After 2h incubation at 37°C, the reaction was stopped by heat treatment (85°C) during 5 min. RT-qPCR was performed in 20μl reaction containing 20 ng of cDNAs, 10 μl of the SYBR PCR master mix (Life Technologies, Fisher) and 400 nM of *tcdA*, *tcdB*, or *tcdR* gene-specific primers (Table S1). (ii) From *in vivo* samples, cDNA from was generated using the reverse transcription protocol for SuperScript VILO Kit (Thermo Scientific, Waltham, MA) without the ezDNase enzyme step. qRT-PCR was performed with 10 μl of the PowerUp SYBR PCR Master Mix (Thermo Scientific, Waltham, MA) and 10 μM of *tcdA*, *tcdB* or *tcdR* gene-specific primers per reaction. A total of 10 ng of template cDNA was added per well with samples run in triplicate. Amplification and detection were performed using a StepOne™ instrument (Applied Biosystem). For each sample, the quantity of cDNAs of target genes was normalized to the quantity of cDNAs of the DNA polymerase III gene (*dnaF*) or the 16S rRNA gene. The relative change in gene expression was recorded as the ratio of normalized target concentrations (threshold cycle [ΔΔ*C*_*T*_] method).^43^

#### Toxin ELISA

Overnight cultures of *C. difficile* strains were diluted in TY medium to a starting OD_600_ of 0.05 and grown at 37°C. 1 ml of culture was harvested after 8, 12 and 24 h growth and centrifuged (5000 rpm at 4°C) for 5 min. Supernatants were collected and kept at -20°C. Cell pellets were washed in PBS 1X and kept at -20°C. For the Toxin A ELISA, PCG4.1 monoclonal antibodies (Bio-techne) used as capture antibodies, were diluted at 4ng/ml in PBS and coated overnight on a Maxisorp plate. The wells were then blocked with Superblock blocking buffer (Thermo Fisher Scientific). A range of purified toxin A (Merck) from 0 to 1 μg/ml used to perform a standard curve and dilutions of the samples were added to the wells. Detection antibodies anti-*C. difficile* toxin A coupled to HRP (LS-Bio) were then added at a 1:10000 dilution. The plate was developed by addition of TMB (Thermo Fisher Scientific) and the reaction was stopped by addition of a 0.2M H_2_SO_4_ solution into the wells. The plate was read at a wavelength of 450nm with a Glomax plate reader (Promega). Toxin concentration in each sample was calculated using the standard curve and normalized by the optical density of the culture. The same procedure is used for the Toxin B ELISA but N4A8 monoclonal antibody (BBI solution) diluted at 4ng/ml in PBS was used for the capture antibody and the T4G1 monoclonal antibody previously coupled to biotin was used as detection antibody (BBI solution, 1:10000 dilution) with streptavidin HRP (Thermo Fisher Scientific). For the CDT ELISA, chicken *C. difficile* binary toxin subunit B capture and detection antibodies (MyBiosource) were used following the supplier’s instructions.

#### Lactate dehydrogenase (LDH) assays

1ml of culture aliquots were taken and pelleted at indicated time points by centrifugation for 5 min at 5000 rpm at 4°C. Supernatants were collected and cell pellets were resuspended in 1 ml of PBS. Resuspended pellets were transferred in Matrix B tubes for lysis by FastPrep (MP biomedicals) with 2x40’’ shaking cycles at speed 6.5, separated by 2 min incubation in ice. LDH activity in the supernatants and the cell lysates was then assessed using the CytoTox 96 Non-Radioactive Cytotoxicity Assay (Promega) by following the manufacturer’s recommendations. Optical density was read at 490nm on a Glomax plate reader (Promega). The ratio of supernatant to total LDH activity normalized by the cell density of the samples was used as an indicator of autolysis.

#### Body condition scoring

Mouse body condition scores (BCS) were assessed on a scale from 1 to 3, with increments denoted by “+” and “-” to indicate intermediate conditions. For example, a BCS of 3- corresponds to a score of 2.66, while a BCS of 2+ corresponds to a score of 2.33. Mouse health was assessed as follows: BCS of 3: The mouse is well-conditioned, with vertebrae and dorsal pelvis not prominent and only palpable with slight pressure. BCS of 2: The mouse is under-conditioned, with noticeable segmentation of the vertebral column and dorsal pelvic bones that are easily palpable. BCS of 1: The mouse is emaciated, with an extremely prominent skeletal structure and distinctly segmented vertebrae. Mice that reached a BCS of 2- were euthanized per IACUC protocol, as this level of disease severity indicated they were unlikely to recover, thus sparing them from further suffering.

#### *In vivo* intracellular vs extracellular toxin ELISA

To separate extracellular and intracellular toxin B fractions, mouse cecal content samples containing *C. difficile* cells were weighed and then resuspended in 1xPBS and then centrifuged at 4°C for 10 minutes at 10,000 × g to pellet bacteria and large debris. The supernatant, containing extracellular toxin B, was carefully collected without disturbing the pellet for Toxin B ELISA. The cell pellet was then washed three times with cold 1xPBS to remove residual extracellular toxins, resuspended in 1ml of cold PBS containing protease inhibitors, and subjected to mechanical lysis using a sonicator with three 10-second pulses on ice to prevent overheating. Following lysis, the sample was centrifuged at 4°C for 10 minutes at 10,000 × g to remove cell debris. The resulting supernatant, containing intracellular toxin B, was collected for ELISA. See Toxin ELISA in STAR Methods for details on how toxin B ELISAs were performed.

#### Histopathological analyses

Formalin-fixed gut segments from germ-free or infected mice were paraffin embedded and 5mm sections cut for staining with hematoxylin and eosin (H&E; Thermo-Fisher, Waltham, MA) as described.^44^ Slides were visualized under a Nikon Eclipse E600 microscope (Nikon, Melville, NY) to assess epithelial damage per cellular stranding and vacuolation, the nature of inflammatory infiltrates, mucosal erosions, and tissue edema. Lumenal neutrophils were quantified by a Pathologist by evaluating ten 400X high powered fields (HPFs) across at least 3 colonic sections per mouse. Neutrophils were identified by presence of segmented nuclei and pale to finely granular cytoplasm.

### Quantification and statistical analyses

Statistical details of experiments, including the number of independent replicates or mice (n) and the statistical tests used, can be found in the figure legends. Briefly, for *in vitro* experiments, data were presented as the mean values ± SEM of at least three independent experiments and the statistical analyses were performed using one-way ANOVA or two-way ANOVA, followed by a Dunnett’s multiple comparisons test or Mann-Whitney test. For all figures, *P* values are represented as follows: ^∗^*P* ≤ 0.05, ^∗∗^*P* ≤ 0.01, ^∗∗∗^*P* ≤ 0.001, ^∗∗∗∗^*P* ≤ 0.0001. For *in vivo* experiments, significant values were evaluated using the Kruskal-Wallis test, a non-parametric statistical test and are represented as follow : ∗∗p=0.0022 and ∗p=0.012.
