# Supplementary material for: Clostridioides difficile’s virulence requires efficient holin-mediated toxin secretion
Source: iScience. 2025 May 7;28(6):112586. doi: 10.1016/j.isci.2025.112586 (PMC12150058; doi:10.1016/j.isci.2025.112586)
Supplement: Document S1. Figures S1–S8 and Tables S1 [file mmc1.pdf]

iScience, Volume *28*

Supplemental information

Clostridioides difficile's virulence requires efficient holin-mediated toxin secretion

Nicholas V. DiBenedetto, Marine Oberkampf, Aline Crouzols, Laura Cersosimo, Vladimir Yeliseyev, Lynn Bry, Johann Peltier, and Bruno Dupuy

Figure S1: TcdA is actively secreted in high toxin producer strains

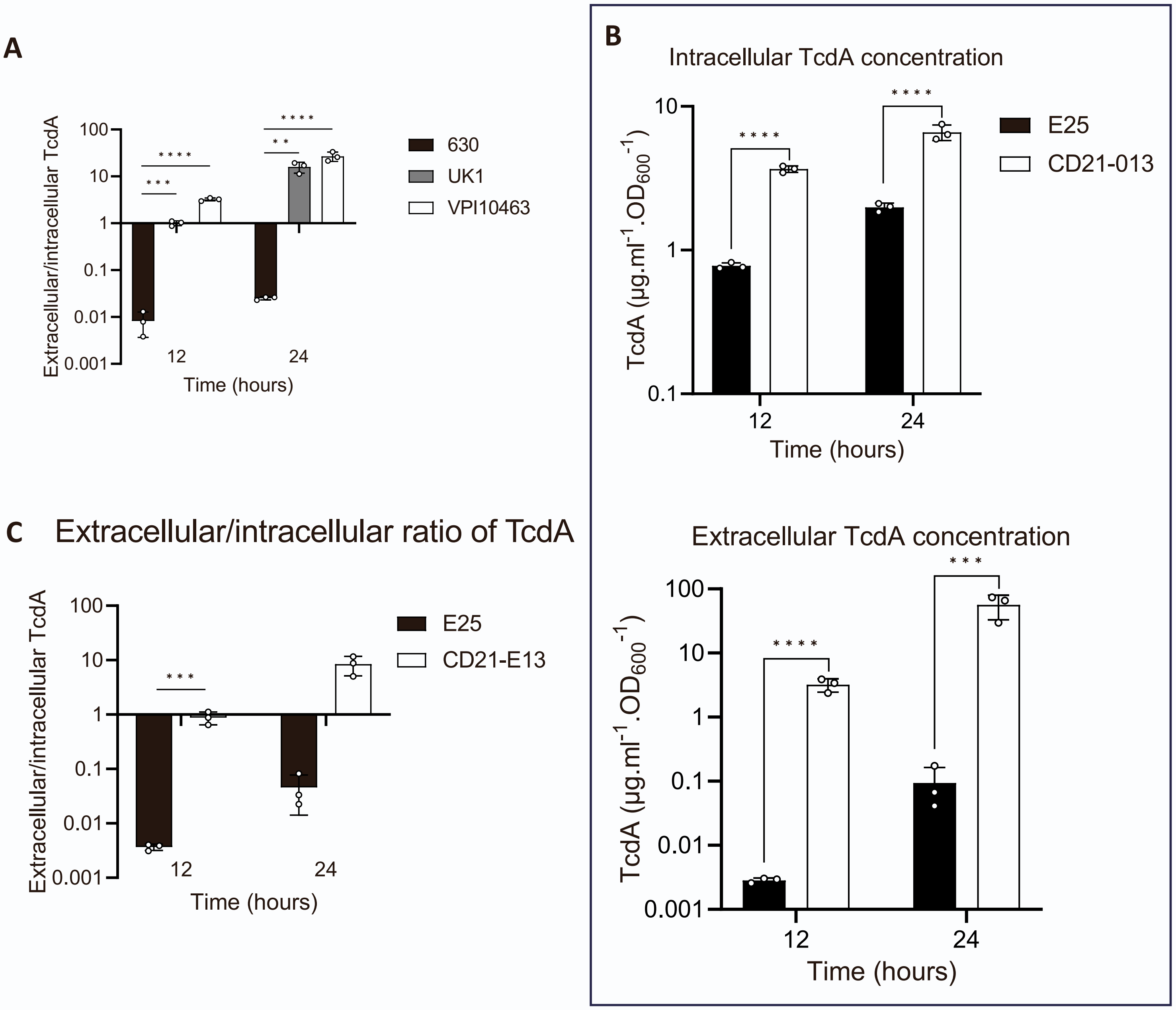

Figure S1: TcdA secretion in low and high-toxin producing strains **A.** extracellular/intracellular ratio of TcdA from 630Δ*erm*, UK1 and VPI10463 strains. The strains were grown in TY medium and TcdA concentrations from the supernatant and the intracellular content were quantified using a TcdA-ELISA. Means and SD are shown; n=3 independent experiments. **p ≤ 0.01, ***p ≤ 0.001 and **** p ≤ 0.0001 by a one-way ANOVA. **B.** TcdA titers in extracellular and intracellular fractions of E25 and CD21-013 strains after 12 and 24 hours of growth. The strains were grown in TY medium and TcdA was quantified using TcdA-ELISA. Means and SD are shown; n=3 independent experiments. ***p ≤ 0.001 and **** p ≤ 0.0001 by a one-way ANOVA. **C.** extracellular/intracellular ratio of TcdA from E25 and CD21-013 strains. The strains were grown in TY medium and TcdA concentrations from the supernatant and the intracellular content were quantified using a TcdA-ELISA. Means and SD are shown; n=3 independent experiments. ***p ≤ 0.001 by a one-way ANOVA

Figure S2: PCR verification of the *ΔtcdE* and *ΔtcdL* mutant strains

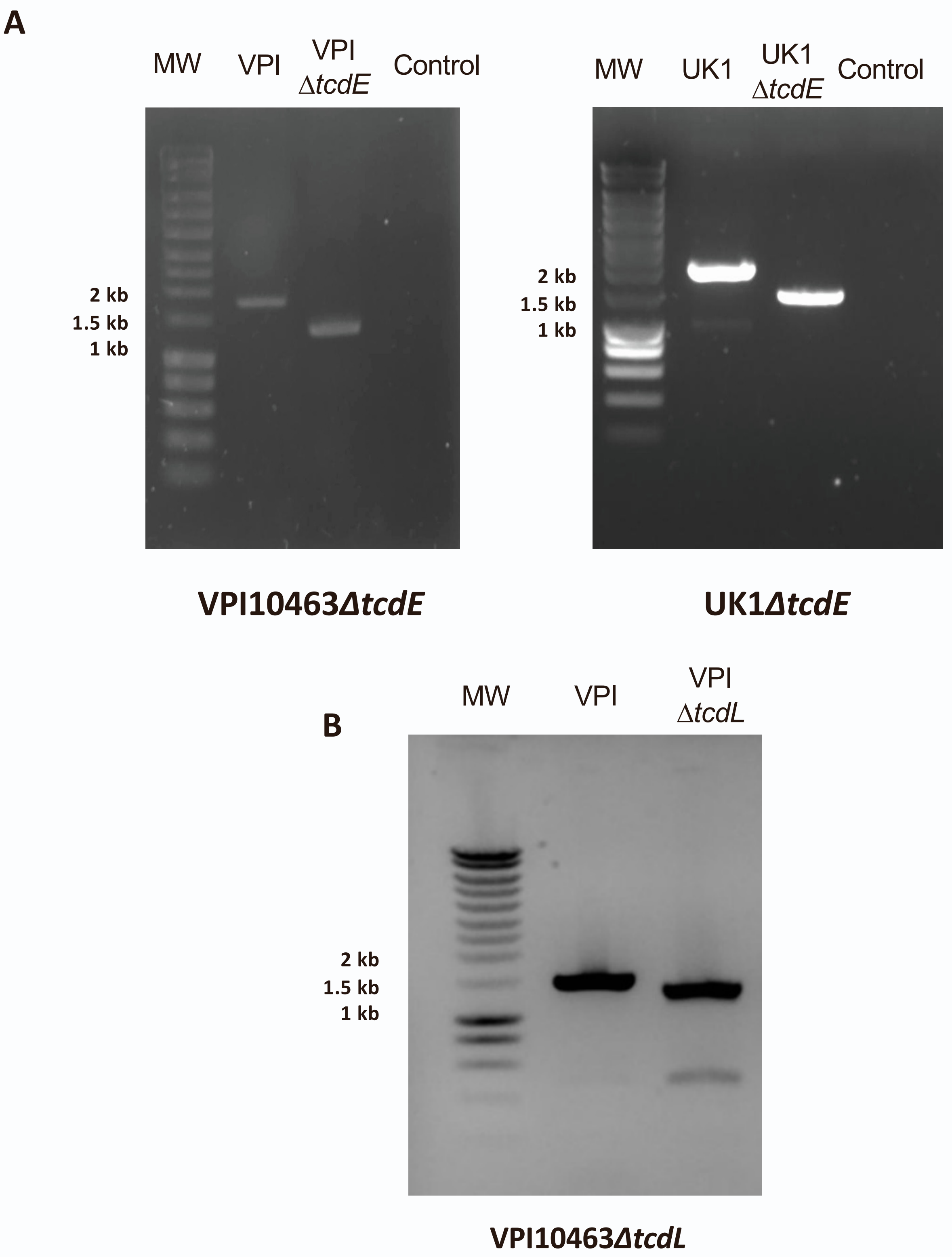

Figure S2: A. PCR verification of the *tcdE* deletion mutants in both C. difficile VPI10463 and UK1 strains. **B.** PCR verification of the *tcdL* deletion mutants in *C. difficile* VPI10463 strain.

Figure S3 : lactate dehydrogenase (LDH) activity in intra- and extracullaire fractions of VPI10463 and UK1 strains and their respective Δ*tcdE* mutants

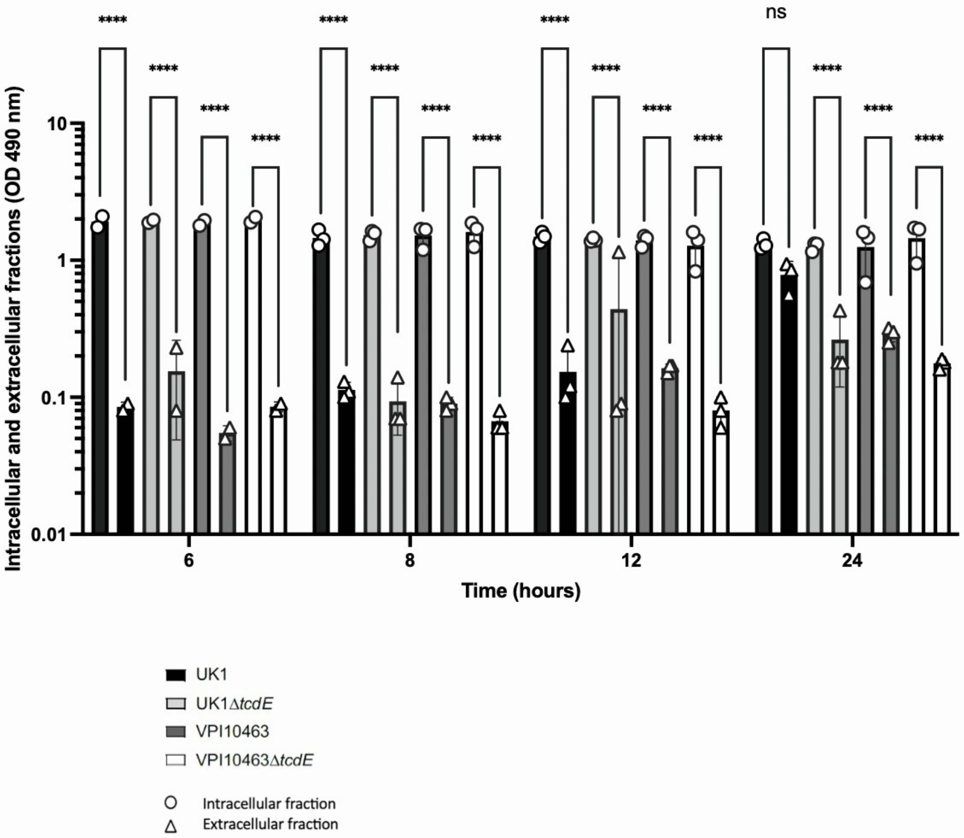

Figure S3: Extracellular and intracellular LDH activity values used to determine the ratio of LDH presented in Fig 1D. Means and SD are shown; n=3 independent experiments. **** $p \leq 0.0001$ by a one-way ANOVA.

Figure S4 : Minor changes of PaLoc genes expression in UK1Δ*tcdE* mutant strain

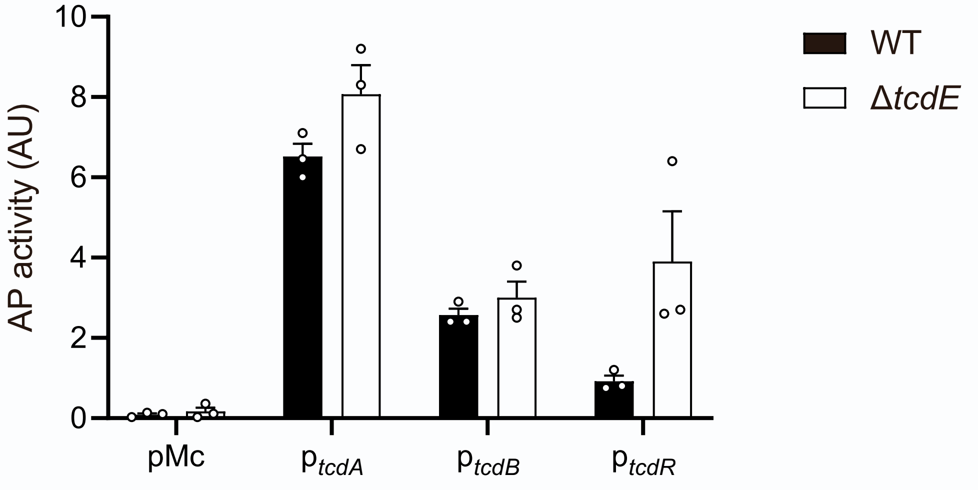

8h UK1

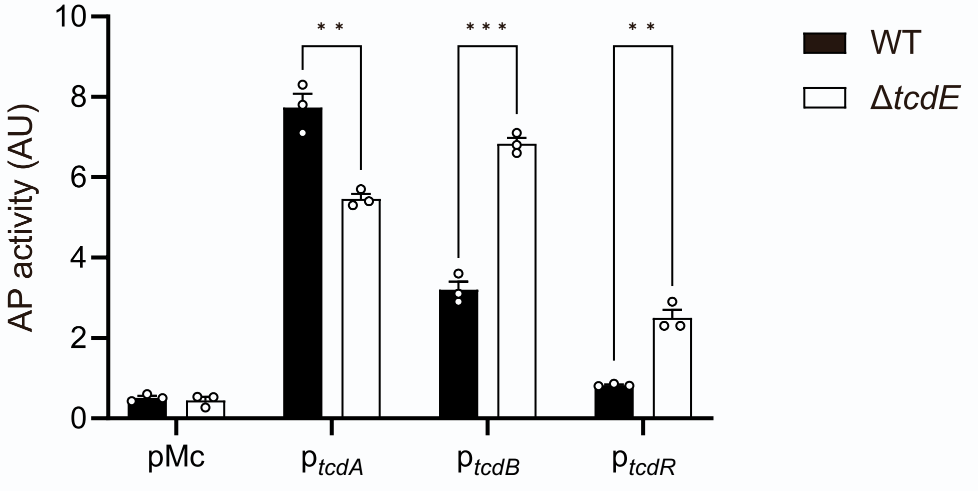

12h UK1

Figure S4: Alkaline phosphatase (AP) activity of P*tcdA*::*phoZ*, P*tcdB*::*phoZ* and P*tcdR*::*phoZ*, expressed from a plasmid in UK1 and UK1Δ*tcdE*. Strains were grown in TY medium and samples assayed for AP activity were collected at 8 and 12 hours of growth. Means and SEM are shown; n=3 independent experiments. **$p \leq 0.01$ and ***$p \leq 0.001$ by an unpaired t test.

Figure S5 : No significant difference in PaLoc gene transcript of the VPI10463 and its isogenic *tcdE* mutant

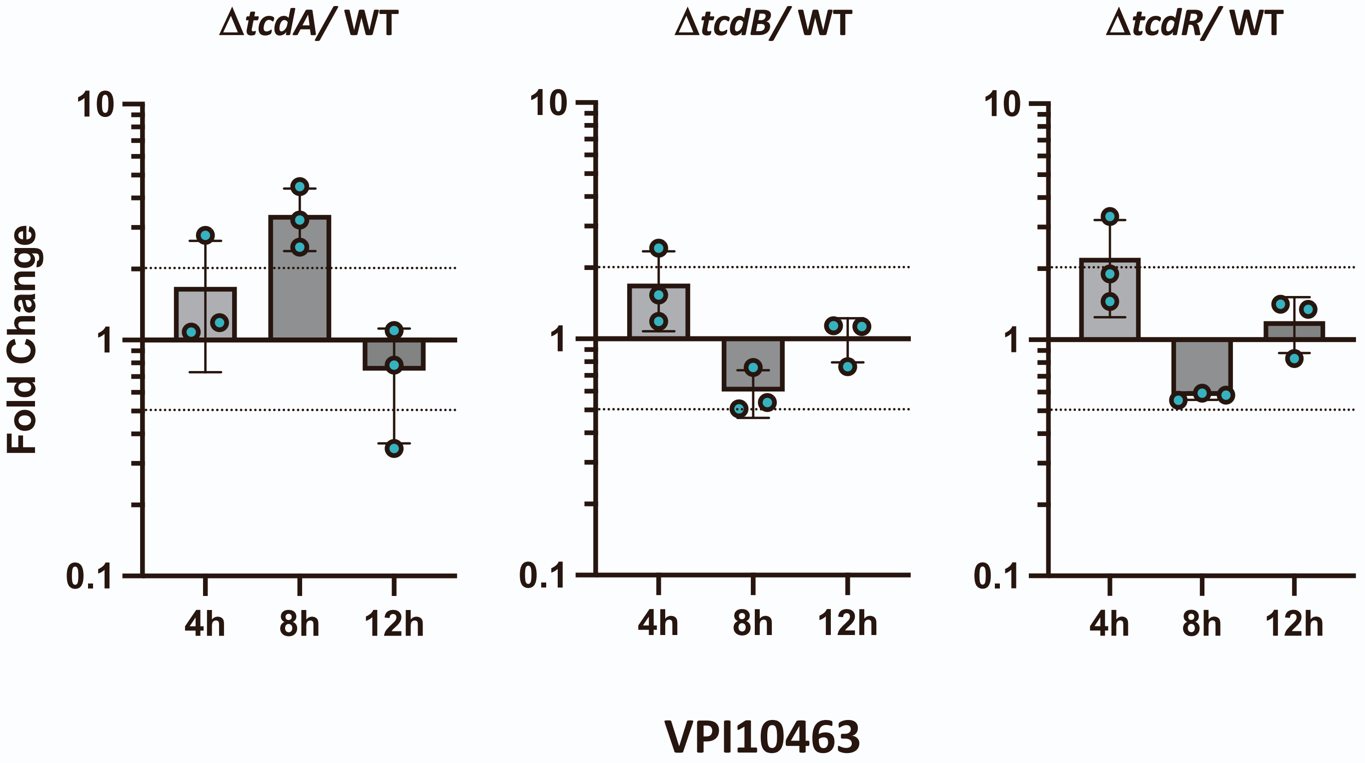

Figure S5:_Transcript abundance of *tcdA*, *tcdB* and *tcdR* quantified by qRT-qPCR. *C. difficile* strains were grown in TY medium. Samples were collected after 4, 8 and 12 hours of growth. Means and SD are shown; n=3 independent experiments.

Figure S6 : TcdL remnant endolysin does not facilitate TcdE-dependent toxin release

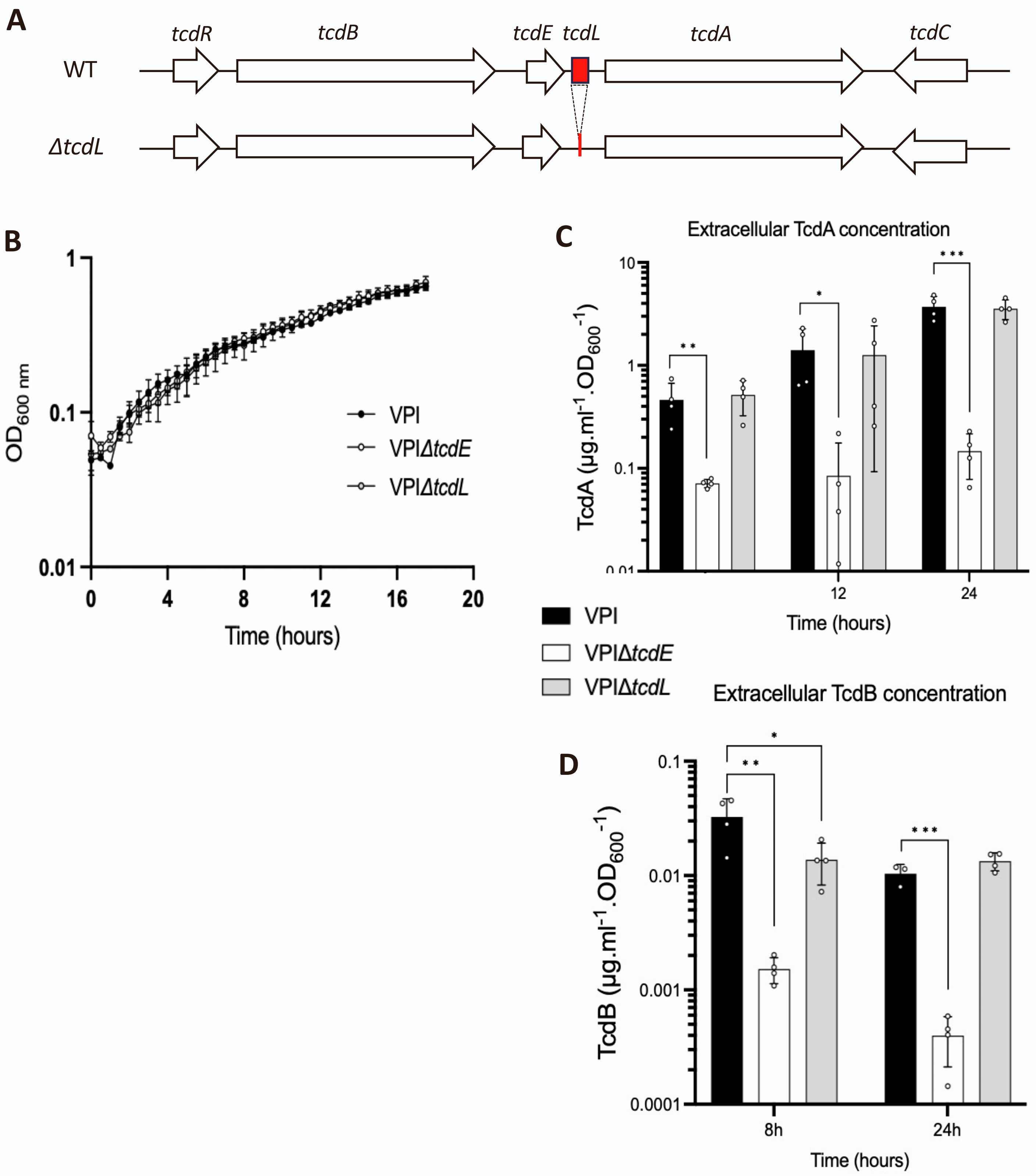

Figure S6: A. Schematic representation of the *tcdL* genetic environment and *tcdL* deletion (Δ*tcdL*). **B.** Growth curves of VPI10463 strain and its respective Δ*tcdE* and D*tcdL* mutants in TY medium. **C.** TcdA (c) and TcdB (d) titers in extracellular fractions of VPI10463 strain and its respective Δ*tcdE* and Δ*tcdL* mutants, after 8, 12 and 24 hours of growth. Strains were grown in TY medium, and toxins were quantified using TcdA- and TcdB-ELISA. Means and SEM are shown; n=4 independent experiments. * p<0,05 ** p<0,01 and *** p<0,001 by a Mann-Whitney test. Horizontal dotted line shows thresholds of detection.

Figure S7 : CDT secretion in the UK1 strain is TcdE-independent

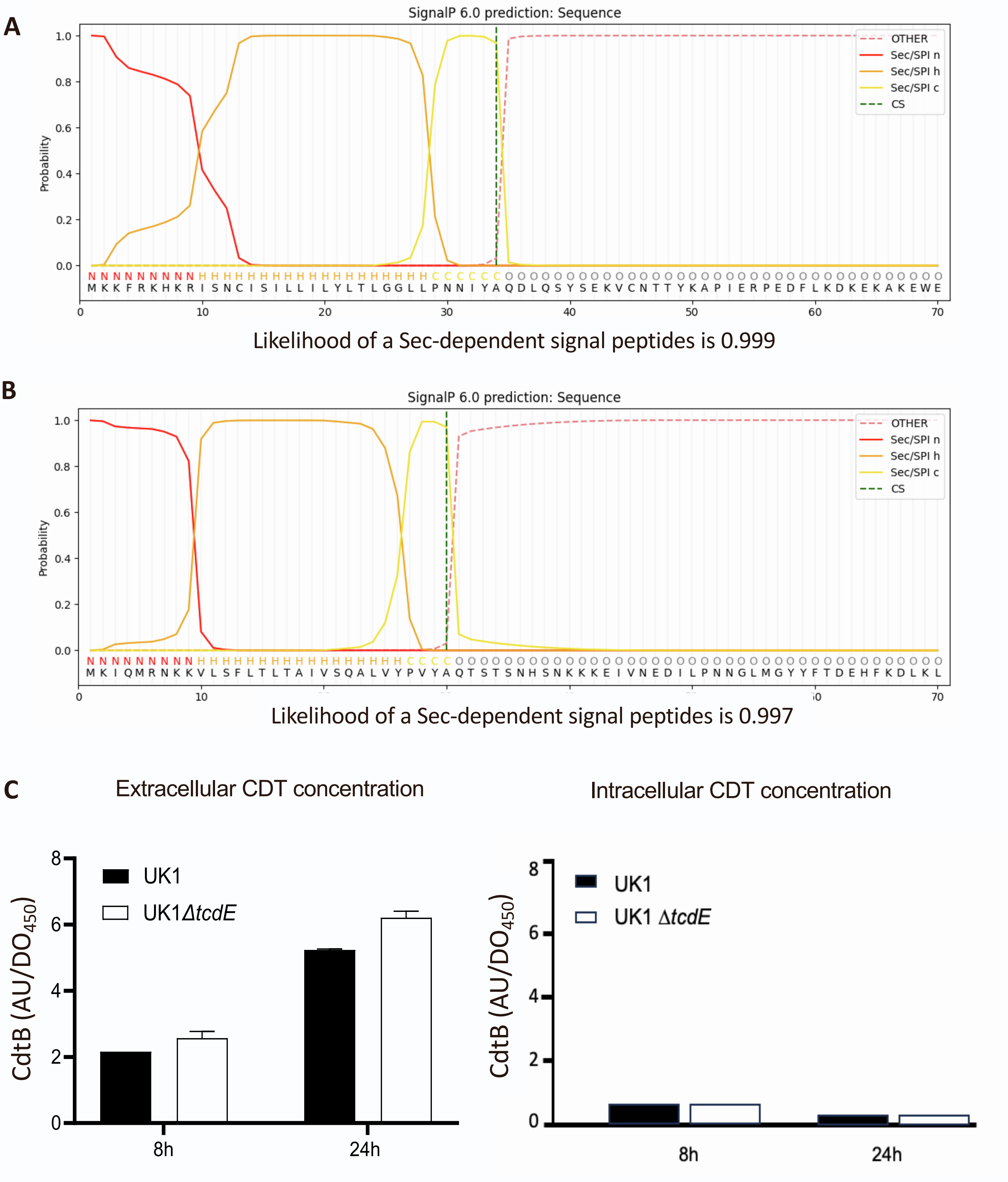

Figure S7: Prediction of a signal peptide from CdtA (**A**) and CdtB (**B**) amino acid sequences using SignalP 6.0 (1). **C**. CdtB levels in extracellular fractions of the UK1 and UK1 Δ*tcdE* strains after 8 and 24h of growth. Strains were grown in TY medium, and CDT was quantified using CDT-ELISA. Means and SD are shown; n=3 independent experiments.

Figure S8 : The UK1 *tcdE* deletion prevent mice from lethal *C difficile* infection

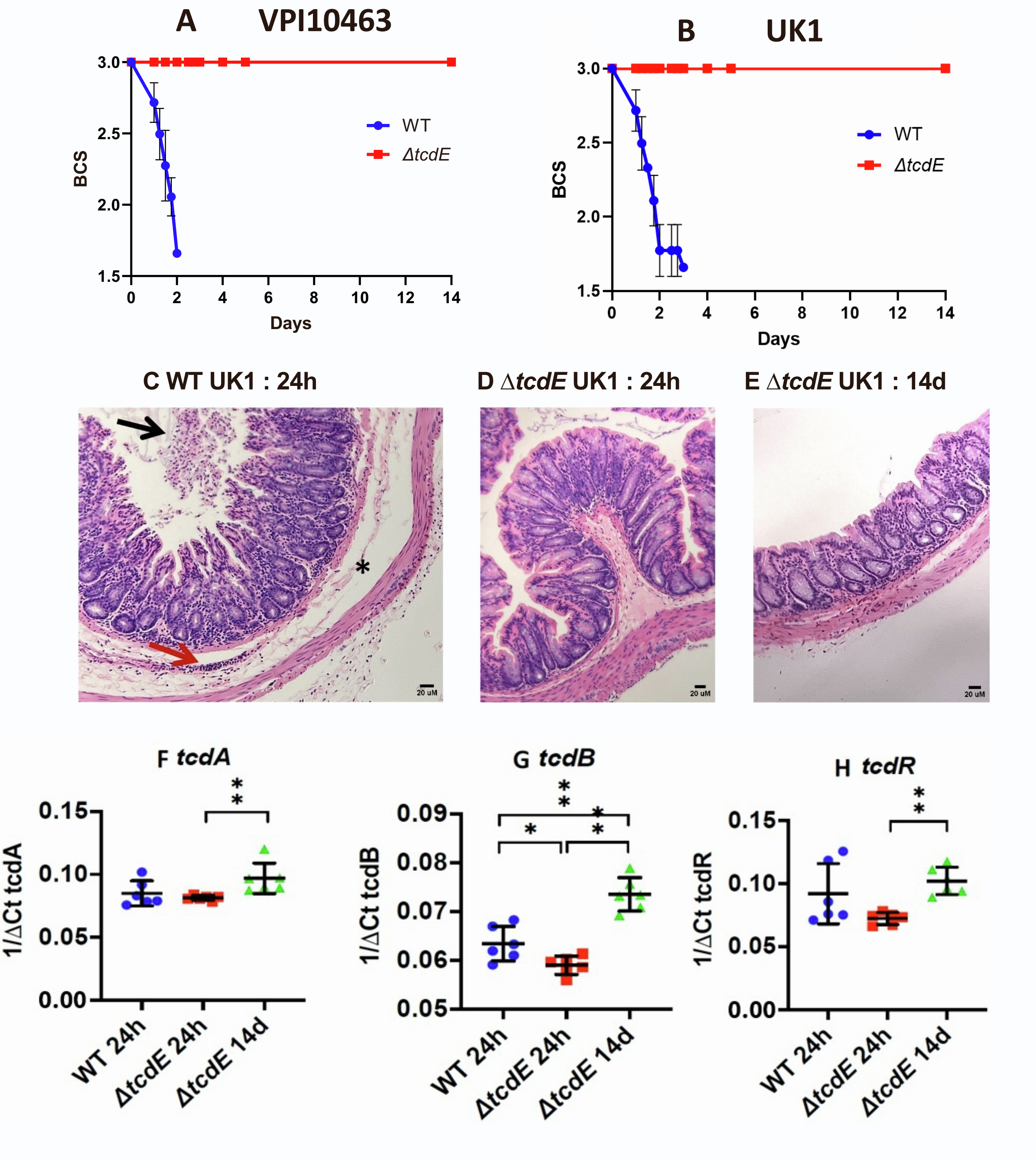

Figure S8: Body condition scores of mice infected with VPI10463 (**A**) and UK1 strain (**B**) and theirs respective *tcdE* mutants. **C-E** Hematolxylin and eosin (H&E) sections of proximal colon from mice infected with wild-type UK1 or D*tcdE* mutant at 200X magnification. **C** Wild-type UK1 infection at 24 hours post-challenge showing transmural inflammation with cellular infiltrates, pseudomembrane formation containing dead epithelial and immune cells (black arrow), underlying sub-mucosal tissue edema (asterix), and influx of inflammatory cells in arterioles entering the sub-mucosa (red arrow). **D** D*tcdE*-infected mouse at 24 h post challenge showing intact mucosa and apical epithelial surfaces, without apparent inflammatory responses or apical colonocyte membrane ruffling from toxin-mediated damage. **E.** D*tcdE*-infected mouse at 14 days post-challenge, showing intact colonic epithelium and mucosa with a mild lymphocytic infiltrate. **F-H** qrtPCR of cecal *tcdA, tcdB* and *tcdR* expression in mice infected with the wild-type or *ΔtcdE* UK1 strains at 24 hours and surviving *ΔtcdE* strain-infected mice at 14 days. Bars show mean and standard deviation.

Table S1. Oligonucleotides used in this study.

Name	Sequence (5'-3')[1]	Description
**pMSR cloning**		
JP449	CGTTTTGTAAACGAATTGC	5’ pMSR0 insert screening
JP450	CTCACGTTAAGGGATTTTG	3’ pMSR0 insert screening
MO-008	TTTTTTGTTACCCTAAGTTTCTCTGACTCTGGAATTATAGAATC	5’ left arm Δ*tcdE* VPI10463
MO-018	CTTTTCATCCAGGTGAACTACTGTGCATTC	3’ left arm Δ*tcdE* VPI10463
MO-019	TAGTTCACCTGGATGAAAAGTAAGTAATGGTAG	5’ right arm Δ*tcdE* VPI10463
MO-020	AGATTATCAAAAAGGAGTTTCTCATTTTCTCTTGGTCTAATG	3’ right arm Δ*tcdE* VPI10463
MO-011	GAATTATGCAAGTTGGATTTG	5’ Δ*tcdE* screening VPI10463
MO-006	CTTTATATATTTTTCCTATAACTTTTAAACTATC	3’ Δ*tcdE* screening VPI10463
MO-021	TTTTTTGTTACCCTAAGTTTGGAATGCAAAATATAGATGATAATTATTTC	5’ left arm Δ*tcdE* UK1
MO-022	CTTACTTTTCGTGCATTCATCATAGTTCAC	3’ left arm Δ*tcdE* UK1
MO-023	ATGAATGCACGAAAAGTAAGTAATGGTAGATATAATAAAAATATTAAC	5’ left arm Δ*tcdE* UK1
MO-024	AGATTATCAAAAAGGAGTTTCGTCTAAATTAGTTAGTATAGTTTTATAC	3’ left arm Δ*tcdE* UK1
MO-025	GGATGGAAGGATTTAGAAGATG	5’ Δ*tcdE* screening UK1
MO-006	CTTTATATATTTTTCCTATAACTTTTAAACTATC	3’ Δ*tcdE* screening UK1
AC001	TTTTTTGTTACCCTAAGTTTCAATTGAGACTGGATGGATATATG	5’ left arm Δ*tcdL* VPI10463
AC002	CTTAATAGATTTTACCCATTATTAGTTTCATG	3’ left arm Δ*tcdL* VPI10463
AC003	GAAACTAATAATGGGTAAAATCTATTAAGC	5’ right arm Δ*tcdL* VPI10463
AC004	AGATTATCAAAAAGGAGTTTCTATTTATTTTTCTCAAAGAATTTG	3’ right arm Δ*tcdL* VPI10463
AC005	GTGGTTTAGTTAGAGTTGGGG	Δ*tcdL* screening VPI10463
AC006	CTATATCATTTTGTCCTTCATCACG	Δ*tcdL* screening VPI10463
**pMC358 cloning**		
JP725	CGTCAATGTATGGGTAGATATG	5’ pMC358 linearization for cloning with Hifi DNA assembly
JP726	CAACGTCGTGACTGGG	3’ pMC358 linearization for cloning with Hifi DNA assembly
JP733	CTATTACGCCAGCTGGC	5’ pMC358-screening
JP734	GGTAACCCCTAGCAAAGC	3’ pMC358-screening
JP758	ttttcccagtcacgacgttgGGTTTCTAGATTTCATAAAAGATAC	5’ *tcdR* promoter
JP759	tatctacccatacattgacgCTCTTATATTTATAATGATGCTTTATTTG	3’ *tcdR* promoter
JP760	ttttcccagtcacgacgttgGTCTGTTTTTGAGGAAGATATTTG	5’ *tcdB* promoter
JP761	tatctacccatacattgacgCTTTACTATAATATTTTTACATCTAAATGC	3’ *tcdB* promoter
JP764	tatctacccatacattgacgCTTTTTATTGGTAAATCTTCATATTAG	5’ *tcdA* promoter
JP765	ttttcccagtcacgacgttgCATGGTCAGTTGGTAAAATC	3’ *tcdA* promoter
***In vitro* qRT-PCR**		
polIII-F_Cdiff	TCCATCTATTGCAGGGTGGT	5' *CD1305*
polIII-R_Cdiff	CCCAACTCTTCGCTAAGCAC	3' *CD1305*
QRTBD013-tcdA	TAATAAAAATACTGCCCTCGACAAA	5’ *tcdA*

QRTBD014-tcdA	ATAAATTGCATGTTGCTTCATAACT	3' *tcdA*
JRP4055	ACCATATAGCTTTGTAGATAGTGAAGGAAA	5' *tcdB*
JRP4056	AAGAACTACATCAGGTAATTCAGATACAAA	3' *tcdB*
QRTBD35-tcdR	AAATAACTCAGTAGATGATTT GCAAGAA	5' *tcdR*
QRTBD36-tcdR	GTTTCTCCCTCTTCATAATGTAAA CT C	3' *tcdR*
**In vivo qRT-PCR**		
tcdA FWD	GCAGTCACTGGATGGAGAATTA	5' *tcdA*
tcdA REV	AGATGATAGCAGTGTCAGGATTG	3' *tcdA*
tcdB FWD	CTGGAGAATGGAAGGTGGTT	5' *tcdB*
tcdB-REV	TTGATGGTGCTGAAAAGAAGTG	3' *tcdB*
BLRT9	CAAGAAATACTCAGTAGATGATTTGCAA	5' *tcdR*
BLRT10	TCTCCCTCTTCATAATGTAAAACTCTA	3' *tcdR*
RT93-16S-F	GGGAGACTTGAGTGCAGGAG	16S
RT94-16S-R	GTGCCTCAGCGTCAGTTACAGT	16S

[1]Lowercase bases indicate overlapping sequences
